# Pediatric Infectious Prepatellar Bursitis with *Kingella kingae*

**DOI:** 10.1155/2020/6586517

**Published:** 2020-01-28

**Authors:** Charles C. Pitts, Walter R. Smith, Michael J. Conklin

**Affiliations:** Department of Orthopaedic Surgery, University of Alabama at Birmingham, USA

## Abstract

We present the first reported case of septic prepatellar bursitis with *Kingella kingae* in a 2-year-old female. Although it is a well-established cause of osteoarticular infections in the pediatric population, *K*. *kingae* has never been reported as the etiology for septic bursitis. A high index of suspicion is required for the diagnosis given that this organism is difficult to culture and isolate using standard laboratory methods. Our diagnosis was established through bursal fluid analysis, though oropharyngeal polymerase chain reaction (PCR) may be also be considered. Our case also builds upon prior literature suggesting that the pathophysiology of septic bursitis in children differs from that of the adult and may be more comparable to that of pediatric osteomyelitis. As an organism of increasing prevalence, *K*. *kingae* should remain high on the differential for osteoarticular or periarticular infections when cultures fail to isolate a distinct pathogen. Early diagnosis and a formal irrigation and debridement, if warranted, are crucial in preventing devastating complications of untreated septic bursitis.

## 1. Introduction

*Kingella kingae* is a Gram-negative bacterium originally described in the 1960s. While clinical interest was minimal for the first thirty years following its discovery, *K*. *kingae* is now recognized as an important pathogen in the pediatric population. It is the leading cause of osteoarticular infections in children aged six to thirty-six months, including septic arthritis and osteomyelitis, with the first series of cases being described by Ceroni et al. and Kiang et al [1, 2].

Oropharyngeal carriage of *K*. *kingae* has previously been shown to have a strong association with osteoarticular infections in children [3]. The cytotoxin RTX (repeat-in-toxin) facilitates spread of the pathogen to the bloodstream and other surrounding tissues [1]. *K*. *kingae* osteoarticular infections usually have a mild presentation. Diagnosis is often challenging, as fever, leukocytosis, positive Gram stain, and elevations in erythrocyte sedimentation rate (ESR) and C-reactive protein (CRP) can be absent. Due to aforementioned reasons, oropharyngeal polymerase chain reaction (PCR) has gained popularity as a useful diagnostic modality when *Kingella* infection is suspected [4, 5]. Although *K*. *kingae* is susceptible to a variety of antibiotics including beta lactams, a high clinical suspicion and early diagnosis are keys in management of the disease [1, 2, 6].

Prepatellar bursitis, or infectious bursitis of any kind, is a relatively rare condition in the pediatric population. One series by Paisley showed that the infection was usually preceded by an injury, traumatic event, or local infection, with *S*. *aureus* and *S*. *pyogenes* being the most common causative organisms [7]. All patients recovered uneventfully when treated with aspiration, incision and drainage, antibiotics, or a combination of these modalities.

We present a case of a *K*. *kingae* infection resulting in prepatellar septic bursitis in a 2-year-old female. Although *K*. *kingae* is a well-established cause of osteoarticular infections in this age group, it has not been reported in the literature as the etiology for a case of septic bursitis.

## 2. Case Report

A 2-year, 5-month-old girl presented to the pediatric orthopaedic clinic for evaluation of left knee pain of about 3 weeks duration. The patient had no medical problems except for an upper respiratory infection approximately 1 month prior to the onset of her symptoms. Reportedly, the patient struck her knee against a bed frame, and this event developed into anterior knee swelling and difficulty bearing weight over the next four days. When her parents noted difficulty with ambulation, she was brought to the emergency department (ED) for evaluation.

**Figure 1:**
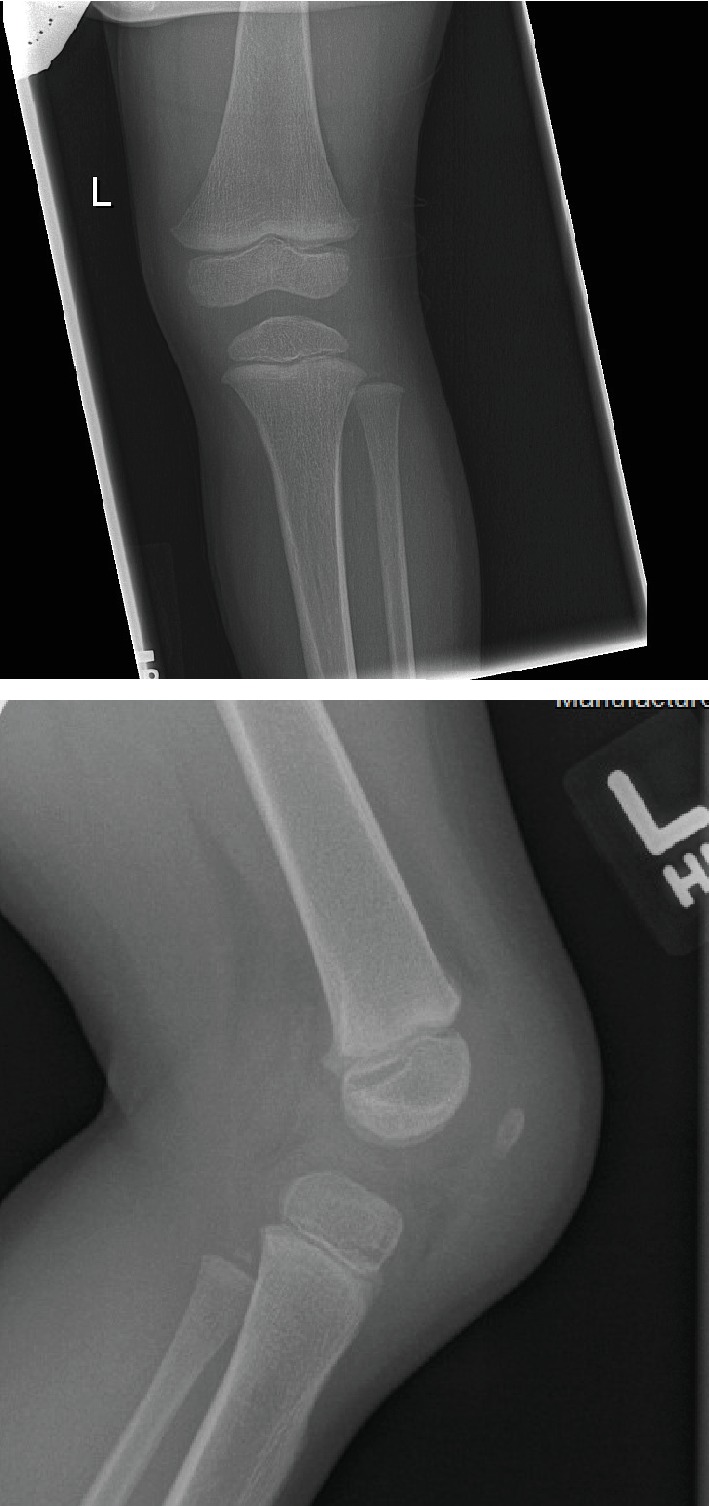
AP and lateral radiograph of the affected knee shows no obvious bony injury or effusion but notable prepatellar soft tissue swelling.

While in the ED, the patient had an erythematous and swollen anterior knee that was warm to the touch. She had pain-free range of motion but walked with a slight limp. She was afebrile with stable vital signs, and parents denied any fever or constitutional symptoms since her injury. Plain radiographs of the left knee were normal with the exception of prepatellar soft tissue edema (Figure 1). Laboratory studies revealed CRP of 0.2 mg/dL (0.0-0.5 mg/dL), ESR 29 mm/hr (0-20 mm/hr), and white blood cell (WBC) count of 6.46 cells × 10^3^ /*μ*L (4.8 − 13.2 × 10^3^/*μ*L). Her symptoms at that time were considered by the ED physician to be posttraumatic with low concern for infectious etiology, and she was discharged without medication.

**Figure 2:**
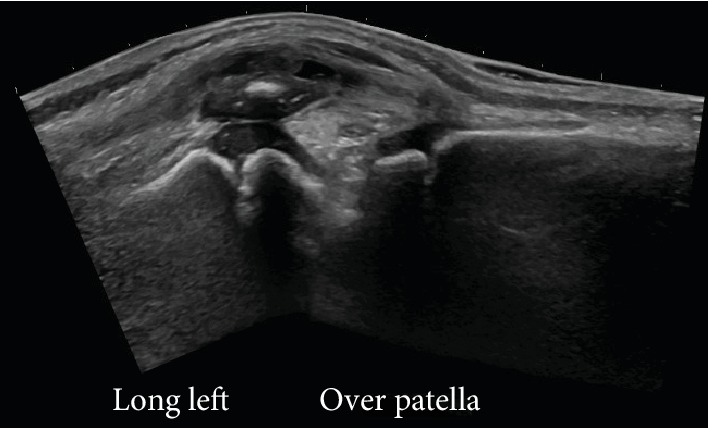
Ultrasound of the affected knee confirms a collection of fluid within the prepatellar bursa.

Her symptoms failed to resolve, and she was evaluated in the orthopaedic clinic two weeks after her ED presentation. She had no constitutional symptoms. She had full passive range of motion of the knee with little pain, but she walked with her left knee held in slight flexion, resulting in a limp. She continued to show swelling and erythema with fluctuance of the prepatellar area that was painful to touch. Her temperature was 98.3 degrees Fahrenheit. Laboratory studies revealed a CRP level of 0.2 mg/dL, ESR 39 mm/hr, and WBC 8.5 cells × 10^3^/*μ*L. An ultrasound of the knee revealed a collection of fluid within the prepatellar bursa (Figure 2). Due to the elevated ESR and prolonged course of symptoms, there was concern for an infectious prepatellar bursitis. Aspiration with possible irrigation and debridement of the prepatellar bursa was recommended.

The patient was brought to the operating room and general anesthesia was induced. Aspiration of the prepatellar bursa yielded thick, blood-tinged, purulent fluid. A small, transverse incision over a Langer's line was made over the prepatellar bursa, and irrigation and debridement were carried out. Bursal fluid was inoculated into a blood culture bottle. Intravenous clindamycin was given, and a stat cell count on the bursal fluid disclosed 74,740 WBC/*μ*L with 83% polymorphonuclear cells (PMNs). Gram stain was negative. The patient was discharged on oral clindamycin.

On postoperative day 3, *Kingella kingae* was isolated from the culture and the patient was called in for follow-up. She was no longer walking with a limp, had full range of motion of the left knee, and had no drainage from her incision. Infectious Disease was consulted and they recommended a 21-day course of oral cephalexin. She was seen again on postoperative day 19 and continued to be asymptomatic and compliant with antibiotic treatment. Her gait was normal, and there was no erythema or fluctuance in the prepatellar area. Repeat ESR, CRP, and WBC count had decreased to within the normal ranges. Radiographs showed no periosteal reaction to suggest osteomyelitis. The patient did not appear for her 6 week follow-up appointment, as her parents had called to inform us that she was having no further symptoms.

## 3. Discussion

While not uncommon in the adult population, septic bursitis is rarely reported in children [7]. There have been few reported cases in the literature, none of which have isolated *K*. *kingae* as the causative organism. Harwell and Fisher reported on ten cases of septic bursitis in children less than sixteen years old, all of which had predisposing trauma similar to our case [8]. However, *Staphylococcus aureus* and *Streptococcus pyogenes* were the only two organisms isolated from the infected bursae [8]. Another series of ten patients over a twenty-five-year span showed similar results with all cultures yielding *S*. *aureus* or *S*. *pyogenes*. The prepatellar bursa was identified as the most common site of occurrence in both series [7, 8].

*Kingella kingae* is particularly evasive of laboratory culture and difficult to isolate using standard methods. An early series described by Yagupsky and Dagan showed growth of *Kingella kingae* on a standard culture medium in only 2 of 25 cases [9]. For this reason, *Kingella* should remain on the differential diagnosis for osteoarticular or periarticular infections even when cultures fail to isolate a distinct pathogen. Therefore, we feel that the current case report is important for delineating *Kingella* as a possible pathogen in pediatric septic bursitis.

The generally accepted pathophysiologic sequence for the development of septic bursitis in adults is repetitive trauma to the area which allows bacterial inoculation through the skin. However, in our case and in previously published series, infection in children is preceded by an acute traumatic event. As previously suggested, this bears resemblance to the pathophysiology of osteomyelitis in the pediatric population. Bacteria leading to osteomyelitis typically spread hematogenously after a minor trauma, suggesting that septic bursitis may develop in a similar fashion. This is also consistent with the fact that *K*. *kingae* is known to disseminate via the bloodstream as opposed to direct invasion through a compromise in the epidermal barrier [10].

The differential diagnosis for a swollen knee in a child is quite broad. Possible etiologies include trauma, intra- or extra-articular infection, and inflammatory arthropathies. A notable physical exam finding that may help distinguish septic arthritis from septic bursitis is severe pain with range of motion, which is generally minimal or absent in septic bursitis. With infection of the prepatellar bursa, there may be pain at extremes of flexion, but generally there is a reasonable arc of motion without pain. Ultrasonography is useful to assess for a fluid collection involving either the joint or the bursa. Routine radiographs should be performed initially and at follow-up to ensure infection of the bone was not missed. Complete blood count with differential and inflammatory markers should be obtained if an infectious etiology is suspected. However, these markers are often insensitive for septic bursitis as demonstrated in our case. Bursal fluid analysis should be sent for laboratory testing. It is important to note that oropharyngeal PCR should be considered if cultures fail to yield an organism, as this has been shown in multiple studies to be predictive of *Kingella kingae* infection [4, 5]. Additionally, it may significantly shorten the time-to-detection. We chose to forego PCR in this scenario due to the fact that bursal fluid yielded an organism within 72 hours.

In summary, we present the first reported case of septic bursitis with *K*. *kingae*. Understanding that *K*. *kingae* may be a cause of septic bursitis is important because it is often difficult to culture. Our case builds upon prior literature suggesting that the pathophysiology of septic bursitis in children differs from that of the adult and may be similar to that of pediatric osteomyelitis. If left untreated, septic prepatellar bursitis may progress to patellar osteomyelitis or septic arthritis of the knee [7]. Early diagnosis and a high index of suspicion is crucial in preventing these complications which may lead to significant bony and cartilaginous destruction.

## References

[1] Ceroni D., Cherkaoui A., Ferey S., Kaelin A., Schrenzel J. (2010). Kingella kingae osteoarticular infections in young children: clinical features and contribution of a new specific real-time PCR assay to the diagnosis. *Journal of Pediatric Orthopaedics*.

[2] Kiang K. M., Ogunmodede F., Juni B. A. (2005). Outbreak of osteomyelitis/septic arthritis caused by Kingella kingae among child care center attendees. *Pediatrics*.

[3] Gravel J., Ceroni D., Lacroix L. (2017). Association between oropharyngeal carriage ofKingella kingaeand osteoarticular infection in young children: a case–control study. *CMAJ*.

[4] Yagupsky P. (2017). Diagnosing Kingella kingae infections in infants and young children. *Expert Review of Anti-Infective Therapy*.

[5] Ceroni D., Dubois-Ferriere V., Cherkaoui A. (2013). Detection of Kingella kingae osteoarticular infections in children by oropharyngeal swab PCR. *Pediatrics*.

[6] Dubnov-Raz G., Ephros M., Garty B. Z. (2010). Invasive pediatric Kingella kingae Infections. *The Pediatric Infectious Disease Journal*.

[7] Paisley J. W. (1982). Septic bursitis in childhood. *Journal of Pediatric Orthopedics*.

[8] Harwell J., Fisher D. (2001). Pediatric septic bursitis: case report of retrocalcaneal infection and review of the literature. *Clinical Infectious Diseases*.

[9] Yagupsky P., Dagan R. (1997). Kingella kingae: an emerging cause of invasive infections in young children. *Clinical Infectious Diseases*.

[10] Yagupsky P. (2015). Kingella kingae: carriage, transmission, and disease. *Clinical Microbiology Reviews*.

